# Role of *MC1R* variants in uveal melanoma

**DOI:** 10.1038/sj.bjc.6601358

**Published:** 2003-11-11

**Authors:** N Hearle, J Humphreys, B E Damato, R Wort, R Talaban, J Wixey, H Green, D F Easton, R S Houlston

**Affiliations:** 1 Haddow Laboratories, Section of Cancer Genetics, Institute of Cancer Research, 15 Cotswold Road, Sutton SM2 5NG, UK; 2 Liverpool Ocular Oncology Centre, Royal Liverpool University Hospital, Liverpool L7 8XP, UK; 3 CRUK Genetic Epidemiology Unit, University of Cambridge, Cambridge, UK

**Keywords:** *MC1R*, uveal melanoma

## Abstract

Variants of the melanocortin-1 receptor (*MC1R*) gene have been linked to sun-sensitive skin types and hair colour, and may independently play a role in susceptibility to cutaneous melanoma. To assess the role of *MC1R* variants in uveal melanoma, we have analysed a cohort of 350 patients for the changes within the major region of the gene displaying sequence variation. Eight variants were detected – V60L, D84E, V92M, R151C, I155T, R160W, R163Q and D294H – 63% of these patients being hetero- or homozygous for at least one variant. Standard melanoma risk factor data were available on 119 of the patients. *MC1R* variants were significantly associated with hair colour (*P*=0.03) but not skin or eye colour. The frequency of the variants detected in the 350 patients was comparable with those in the general population, and comparison of the cumulative tumour distribution by age at diagnosis in carriers and noncarriers provided no evidence that *MC1R* variants confer an increased risk of uveal melanoma. We interpret the data as indicating that *MC1R* variants do not appear to be major determinants of susceptibility to uveal melanoma.

Uveal melanoma although rare is the most common primary intraocular malignancy in adults with an incidence of six per million per year (Parkin *et al*, 1992). Compared to cutaneous melanoma, little is known about the aetiology of the disease. Recognised risk factors for cutaneous melanoma include pale skin and fair hair, number of naevi, atypical naevi, tendency to freckle and sensitivity to sunlight (Bliss *et al*, 1995). While both cutaneous and uveal melanomas develop from melanocytes originating in the neural crest and also share several histological characteristics, there are distinctive differences between the two types of tumour in terms of cytogenetic anomalies and familial inheritance.

Unlike cutaneous melanoma, the genetic predisposition to uveal melanoma has not been studied extensively. While segregation of uveal melanoma and cutaneous melanoma and an association with atypical melanoma have been reported (Bataille *et al*, 1993; van Hees *et al*, 1998), most of the uveal melanomas appear to be sporadic (Lynch *et al*, 1968; Canning and Hungerford, 1988; Singh *et al*, 1996). Several studies have suggested that atypical naevi, light eye colour and exposure to ultraviolet radiation each represent independent risk factors for uveal melanoma, just as they do for cutaneous melanoma (Rootman and Gallagher, 1984; Dolin and Johnson, 1994; Bataille *et al*, 1995; Regan *et al*, 1999). The inference from these data is that genetic susceptibility to uveal melanoma is likely to be mediated through sensitivity to ultraviolet radiation.

Melanin pigmentation plays an important role in affording protection against the deleterious effects of ultraviolet radiation. In humans, *α*-melanocyte-stimulating hormone (*α*-MSH) and other pro-opiomelanocortin peptides modulate melanin pigment formation via the melanocortin-1 receptor (MC1R) on melanocytes (Sturm *et al*, 2001). Two melanin pigments have been identified in humans: black eumelanin and red phaeomelanin. The relative proportions of these pigments are controlled by *α*-MSH via MC1R. Eumelanin is protective, but phaeomelanin may contribute to carcinogenesis through production of free radicals (Ranadive *et al*, 1986; Sturm, 1998). Several point mutations in *MC1R* affecting function have been identified, for example, V60L, R151C, R160W, D294H, some of which have been reported to be over-represented in individuals with fair hair and skin (Valverde *et al*, 1995; Flanagan *et al*, 2000; Box *et al*, 2001a,2001b). In addition to acting as determinates of pigmentation, some variants may confer an increased risk of cutaneous melanoma (Valverde *et al*, 1996; Healy *et al*, 1999; Palmer *et al*, 2000). *MC1R* genotype has been shown to have a persisting effect on risk of cutaneous melanoma and nonmelanoma skin cancer even after adjusting for hair and skin colour, which supports the notion that MC1R may directly modulate melanocyte growth and differentiation (Valverde *et al*, 1996).

We have assessed the risk of uveal melanoma associated with germline *MC1R* variants through sequence analysis of 350 patients and a series of 133 population controls. We have also evaluated the role of these variants as determinants of skin type, hair and eye colour, and cutaneous naevus count in 119 of the patients.

## PATIENTS AND METHODS

### Patients

A total of 350 unrelated patients with uveal melanoma attending the Liverpool Ocular Oncology Centre (LOOC) between 1994 and 1997, either for treatment of a newly diagnosed tumour (*n*=300) or for review after previous treatment (*n*=50), were studied. In all, 183 of the patients were male (52%). The average age at diagnosis of uveal melanoma was 58 years (s.d. 13, range 22–89). The diagnosis of uveal melanoma was based on ophthalmoscopy and ultrasonography performed by an experienced examiner (BD) and, in patients treated by enucleation or local resection, was confirmed by histology. There was no selection of patients. The only exclusion criterion was being nonwhite.

Standard risk factors for cutaneous melanoma, propensity to sunburn, pigmentation (skin colour, hair colour at 15 years, eye colour) and number of cutaneous naevi were collected by interview and clinical examination in 119 consecutive patients aged 18–60 years. Hair colour was classified into: red, auburn, blond/fair, light brown, medium brown, dark brown and black. A three-point scale was used for eye colour, using the categories ‘blue’, ‘green/grey’ and ‘hazel/brown’. Skin type was classified using an extension of Fitzpatrick's scheme (1988) as follows: type I, individuals who always burn and never tan; type II, always burn then tan slightly (mild tanning); type III, sometimes burn and always tan (moderate tanning); type IV, burn minimally and tan easily; type V, rarely burn and tan deeply; type VI, intense tanning with no burning.

Blood samples from 133 healthy spouses of colorectal cancer cases served as controls – 53 males and 80 females, mean age 56 years (s.d. 9, range 30–89). None of the controls had a personal history of malignancy. All were Caucasian and their ancestry was from the British Isles.

Blood samples and clinical data were obtained with informed consent and Local Ethical Review Board approval in accordance with the tenets of the Declaration of Helsinki.

### Molecular analyses

DNA was extracted from EDTA venous blood samples using a standard sucrose lysis protocol. Detection of sequence variation in *MC1R* nucleotides 107–492 was undertaken by sequencing. Two sets of overlapping primers M1F 5′-AGCCCGGTGCCTGGAGGTGT-3′ and M1R 5′-TGGTAGCGCAGTGCGTAGAA-3′ and M2F 5′-GGGAGCAAC(GA)TGCTGGAGAC-3′ and M2R 5′- ACCGGGCGCTGCCTCTTGTG-3′ were used and sequences determined using the ABI Ready Reaction Dye Terminator Cycle Sequencing Kit and ABI377 or ABI3100 semiautomated sequencers. Sequence analysis was undertaken using Sequence Navigator Software (PE Applied Biosystems, Foster City, CA, USA), and nucleotide changes identified in *MC1R* were referenced to the published sequence (Genbank accession number NM_002386). The D294H variant was detected using a polymerase chain reaction restriction fragment length polymorphism. A 330 bp fragment of *MC1R* was amplified by PCR using the primers: M3F, 5′-ACCATCCTGCTGGGCATTTT-3′ and 5′-ACGGGGACCAGGGAGGTAAG-3′. The PCR product was digested with *Taq*I in accordance with the manufacturers’ recommendations (New England Biolabs Inc., Beverly, MA, USA). The G-allele (D294) is cleaved generating 150 and 180 bp fragments cleavage whereas the A-allele (294H) is refractory to cleavage. Cleavage products were visualised on 2% agarose gels and digest products confirmed by sequencing. Two researchers confirmed sequence and genotyping analyses.

### Statistical analyses

All statistical analyses were performed using the statistical software program STATA Version 7 (Stata Corporation 702 University Drive East, College Station, TX 77840, USA. http://www.stata.com). The distribution of categorical variables was compared by either *χ*^2^ or Fisher's exact test. ANOVA or *t*-test was used to test differences in the distribution of normally distributed continuous variables. Naevi counts were log transformed to normalise their distribution. Carrier and noncarrier cumulative tumour distributions by age at diagnosis were compared using log-rank test. The relationship between *MC1R* genotype and risk of uveal melanoma was assessed by means of the odds ratio (OR) with 95% confidence limits calculated by unconditional logistic regression adjusting for age and sex. A test for trend (*P*_trend_) in increasing the risk of uveal melanoma by having more than one putative high-risk allele of *MC1R* was also evaluated. Pooled estimates of the OR for this and previously published studies were obtained by calculating a weighted-average of the logarithm of ORs (Breslow and Day, 1987). Studies were weighted according to the inverse of the variance of the log of the OR. A *P*-value of 0.05 was considered statistically significant.

## RESULTS

Eight variants were detected – V60L, D84E, V92M, R151C, I155T, R160W, R163Q, D294H – among the patients and controls studied (Table 1

**Table 1:** Relationship between *MC1R* variants and risk of uveal melanoma in this study

	**Cases**	**Controls**	**OR^a^**	**95% CI^a^**
*Number of MC1R variants*				
0	129/350 (36.9%)	50/133 (37.6%)	1.0	—
1	154/350 (44.0%)	55/133 (41.4%)	1.09 (1.09)	0.69–1.70 (0.70–1.72)
2+	67/350 (19.1%)	28/133 (21.0%)	0.93 (0.96)	0.54–1.61 (0.55–1.68)
Any variant	221/350 (63.1%)	83/133 (62.4%)	1.03 (1.05)	0.68–1.56 (0.69–1.60)
				
*Frequency of MC1R variant*				
V60L	77/350 (22.0%)	36/133 (27.3%)	0.73 (0.76)	0.44–1.15 (0.48–1.22)
D84E	9/350 (2.6%)	4/133 (3.0%)	0.67 (0.60)	0.22–2.05 (0.19–1.89)
V92M	62/350 (17.7%)	21/133 (15.8%)	1.15 (1.09)	0.67–1.97 (0.62–1.89)
R151C	50/350 (14.2%)	19/133 (14.4%)	1.00 (0.98)	0.57–1.77 (0.55–1.76)
I155T	3/350 (0.9%)	1/133 (0.8%)	1.14 (0.75)	0.12–11.07 (0.07–7.52)
R160W	48/350 (13.7%)	15/133 (11.4%)	1.16 (1.23)	0.63–2.13 (0.66–2.29)
R163Q	22/350 (6.3%)	12/133 (9.1%)	0.68 (0.78)	0.32–1.41 (0.37–1.68)
D294H	19/350 (5.4%)	4/133 (3.0%)	1.85 (1.88)	0.62–5.55 (0.61–5.78)

a OR and 95% CI values adjusted for age and sex are given in parentheses.

). The frequencies of combinations of *MC1R* variants did not differ from expected observed allele frequencies (data not shown). Hence, the *MC1R* variants can be considered as independent variants and consequently were analysed as such.

The frequencies of the *MC1R* variants that have been reported in 190 UK population controls – D84E (3.5%), V92M (17.3%) and D294H (6.8%) (Ichii-Jones *et al*, 1998) – are not statically different from that observed in the cases and controls in our study. In addition, the frequencies of the V60L, D84E, R151C, R160W and D294H variants are not statistically different from the frequencies previously documented in the 738 individuals of Northern European ancestry reported by Vajdic *et al* (2003) (23.2, 2.8, 20.6, 15.6 and 6.0%, respectively).

Information on hair colour, eye colour and skin type had been recorded in 119 of the patients. There was a strong relationship between skin and fair hair and eye colour (*P*<0.05). The relationship between these three phenotypes and *MC1R* variation is detailed in Table 2

**Table 2:** Numbers of homo- and heterozygotes for *MC1R* alleles in patients according to skin type, and hair and eye colour

	***MC1R* variant^a^**								
**Phenotype**	**V60L**	**D84E**	**V92M**	**R151C**	**I155T**	**R160W**	**R163Q**	**D294H**	**Combined**
*Skin type*									
I (*n*=13)	1	1	3	2	0	2	3	2	10
II (*n*=38)	6	0	7	7	0	11	2	2	26
III (*n*=29)	7	0	6	3	0	5	2	1	20
IV (*n*=25)	3	1	8	3	0	3	1	1	16
V (*n*=12)	3	1	2	1	0	0	0	0	6
VI (*n*=2)	0	0	0	0	0	0	0	0	0
									
*Hair colour*									
Red (*n*=2)	0	1	0	0	0	0	0	1	2
Auburn (*n*=7)	2	0	0	1	0	3	1	2	6
Blond (*n*=16)	2	0	3	8	0	3	2	2	15
Light brown (*n*=26)	3	0	7	1	0	4	1	1	14
Medium brown (*n*=20)	4	0	7	2	0	4	0	0	14
Dark brown (*n*=35)	6	0	7	3	0	4	3	0	20
Black (*n*=13)	2	2	2	1	0	2	0	0	7
									
*Eye colour*									
Blue (*n*=68)	10	0	17	11	0	8	5	3	44
Green-grey (*n*=12)	2	0	2	0	0	4	0	1	7
Hazel-brown (*n*=39)	9	3	7	5	0	9	3	2	28

a Compound heterozygotes appear in more than one column under the analysis of individual alleles, but only once in the combined column. Thus, the “combined” column does not necessarily represent the sum of values for the individual alleles.

. No significant association was seen between possession of one or more *MC1R* variants and eye colour (*P*=0.46). Similarly, no significant relationship was seen between skin type and possession of a *MC1R* variant (*P*=0.29) or naevus count (*P*=0.45). There was, however, an association between *MC1R* status and hair colour with an over-representation of variants in individuals with light or red hair (*P*=0.03).

The frequency of each of the *MC1R* variants in the 350 patients was not statistically different from that observed in the controls. Of the 350 patients studied, 129 (36.9%) had no *MC1R* variants, 154 (44.0%) had one *MC1R* variant and 67 (19.1%) possessed two or more variants.

The mean ages at diagnosis in noncarriers and carriers of one and two or more of *MC1R* variants in our study were not significantly different – 57.9, s.d. 14; 58.0, s.d. 13 and 58.0, s.d. 12, respectively; *P*=0.99. Similarly, the cumulative distributions of age at diagnosis in carriers of one and two or more variants, and noncarriers were not significantly different.

Two research groups have previously reported on the relationship between MC1R variants and risk of uveal melanoma – Metzelaar-Blok *et al* (2001) based on analysis of 162 patients and Vajdic *et al* (2003) based on analysis of 62 patients. In both studies, there was no difference in the frequency of variants in cases compared to controls. Table 3

**Table 3:** Estimates of risk of uveal melanoma associated with *MC1R* variants in this and published studies and meta-analyses of studies

	**Current study**		**Metzelaar-Blok *et al* (2001)**		**Vajdic *et al* (2003)**		**Pooled data^a^**	
	**OR^a^**	**95% CI**	**OR**	**95% CI**	**OR**	**95% CI**	**OR**	**95% CI**
*Number of MC1R variants*								
0	1.0	—	1.0	—	—	—	1.0	—
1	1.09 (1.09)	0.69–1.70 (0.70–1.72)	0.83	0.53–1.30	—	—	0.95	0.69–1.30
2+	0.93 (0.96)	0.54–1.61 (0.55–1.68)	1.01	0.59–1.77	—	—	0.99	0.67–1.46
Any	1.03 (1.05)	0.68–1.56 (0.69–1.60)	0.88	0.58–1.34	1.05	0.19–1.59	0.96	0.71–1.29
								
*Specific MC1R variant*								
V60L	0.73 (0.76)	0.69–1.70 (0.70–1.72)	1.06	0.64–1.75	1.47	0.85–2.45	1.02	0.77–1.35
D84E	0.67 (0.60)	0.22–2.05 (0.19–1.89)	2.39	0.39–14.44	0.44	0.02–2.43	0.80	0.33–1.99
V92M	1.15 (1.09)	0.67–1.97 (0.62–1.89)	1.06	0.63–1.179	—	—	1.07	0.81–1.40
D142H	—	—	1.58	0.22–11.34	—	—	—	—
R151C	1.00 (0.98)	0.57–1.77 (0.55–1.76)	1.08	0.58–2.00	0.67	0.33–1.28	0.91	0.64–1.30
I155T	1.14 (0.75)	0.12–11.07 (0.07–7.52)	0.52	0.053–5.06	—	—	0.62	0.12–3.18
R160W	1.16 (1.23)	0.63–2.13 (0.66–2.29)	1.08	0.67–1.73	1.09	0.52–2.14	1.12	0.81–1.57
R163Q	0.68 (0.78)	0.32–1.41 (0.37–1.68)	0.95	0.48–1.91	—	—	0.87	0.52–1.45
P230L	—	—	2.12	0.47–9.63	—	—	—	—
H260P	—	—	6.43	0.71–58.05	—	—	—	—
D294H	1.85 (1.88)	0.62–5.55 (0.61–5.78)	6.43	0.71–58.05	0.87	0.19–2.71	1.67	0.75–3.72

a Based on pooling adjusted estimates from the current study.

summarises the results from these two studies. Also shown are pooled estimates of the risk of uveal melanoma based on all studies for individual variants and one, two or more and any *MC1R* variant. In pooling studies, there was no evidence of heterogeneity. No statistically significant associations between risk and MC1R status were detected in this analysis.

## DISCUSSION

We performed this study to determine whether germline *MC1R* variants confer an increased risk of uveal melanoma. Our investigation was prompted by the observation that patients with uveal melanomas have a greater number of cutaneous naevi than that of the general population, and that numbers of cutaneous naevi, as well as skin and hair colour, are a function of *MC1R* genotype (Bataille *et al*, 1995).

The frequency of *MC1R* variants detected in patients in our study was not significantly different from the frequencies in the general population, and were very similar to estimates obtained in unselected North-European populations. Furthermore, we found that the age at diagnosis of uveal melanoma in carriers of *MC1R* variants was not significantly different from noncarriers. These findings imply that *MC1R* variants are unlikely to confer an increased risk of uveal melanoma. The main strengths of our study are the large number of patients in our sample and correlation of *MC1R* variants with age at diagnosis, a factor that has not previously been investigated.

In our study, we detected eight *MC1R* variants: V60L, D84E V92M, R151C, I155T, R160H, R163Q and D294H. Linkage disequilibrium has been reported to exist between certain variants (Palmer *et al*, 2000). In our study this was nondetectable; however, our study was not empowered to explore this. Previous studies have demonstrated a relationship between *MC1R* variants and hair and skin type, notably a strong association between the R151C, R160Q and R294H variants, with fair skin and red hair (Valverde *et al*, 1995; Palmer *et al*, 2000; Bastiaens *et al*, 2001). Collectively, the *MC1R* variants we detected were over-represented in the patients with light skin and red or fair hair, in keeping with these previous observations. The relationship was, however, less pronounced than in some previous studies, largely because of the small number of patients with red or auburn hair and type I skin type in our study.

Most, but not all, previous studies have shown that *MC1R* variants are associated with an increased risk of both cutaneous melanoma (Valverde *et al*, 1996; Palmer *et al*, 2000) and nonmelanoma skin cancers (Box *et al*, 2001a,2001b). There is some evidence that this effect persists even after correcting for skin type. This suggests that certain *MC1R* variants can exert an effect on melanoma tumorigenesis in a dual manner, both as a determinant of fair skin and as a component in an independent additional pathway (Palmer *et al*, 2000; Van der Velden *et al*, 2001).

Our data support the findings of Metzelaar-Blok *et al* (2001) and the recent study reported by Vajdic *et al* (2003), who found no relationship between variation in *MC1R* and risk of uveal melanoma. Moreover, pooling data from all three studies provide no evidence that variants confer an increased risk of uveal melanoma. We cannot, however, exclude the possibility that variants confer very small increases in risk of ∼1.1-fold.

In our study, the majority of patients had blue eyes in keeping with the finding of Regan *et al* (1999), who reported that uveal melanoma is more common in individuals with light irises, suggesting that increased sensitivity to sunlight from prolonged ultraviolet exposure represents a risk factor. Hence, our findings do not necessarily contradict the observation that ultraviolet radiation and pigmentation probably represent risk factors for uveal melanoma. However, in contrast to cutaneous melanoma, most of the patients with uveal melanomas in our study and other reports (Metzelaar-Blok *et al*, 2001) do not have type I or type II skin.

Although of a common embryological origin, there are several biological differences between uveal, conjunctival and cutaneous melanocytes (Sarna, 1992). Firstly, there is no evidence that ultraviolet light initiates melanogenesis in uveal melanomas (Sahm *et al*, 2001). Secondly, it is unclear whether uveal melanocytes continue to synthesise melanin in adulthood (Hu *et al*, 1995). Lastly, whereas epidermal melanocytes synthesise melanosomes and export these to keratinocytes, melanosomes within uveal melanocytes remain relatively inactive (Boissy, 1988). These differences may explain at least in part why incidence rates of uveal melanoma do not show such geographical differences to those seen in cutaneous melanoma. Moreover, differences in the relative importance of aetiological factors between the two tumour types are reflected at the molecular level – activating mutations of *BRAF* are almost universal in cutaneous melanomas but not in uveal tumours (Davies *et al*, 2002; Cohen *et al*, 2003; Edmunds *et al*, 2003).
